# Supplementary material for: Photo-thermo-induced room-temperature phosphorescence through solid-state molecular motion
Source: Nat Commun. 2022 Jul 6;13:3887. doi: 10.1038/s41467-022-31481-3 (PMC9259671; doi:10.1038/s41467-022-31481-3)
Supplement: Supplementary file 1 — Supplementary Information [file 41467_2022_31481_MOESM1_ESM.pdf]

Synthetic route of *p*-Br-TRZ, *m*-Br-TRZ, and *o*-Br-TRZ

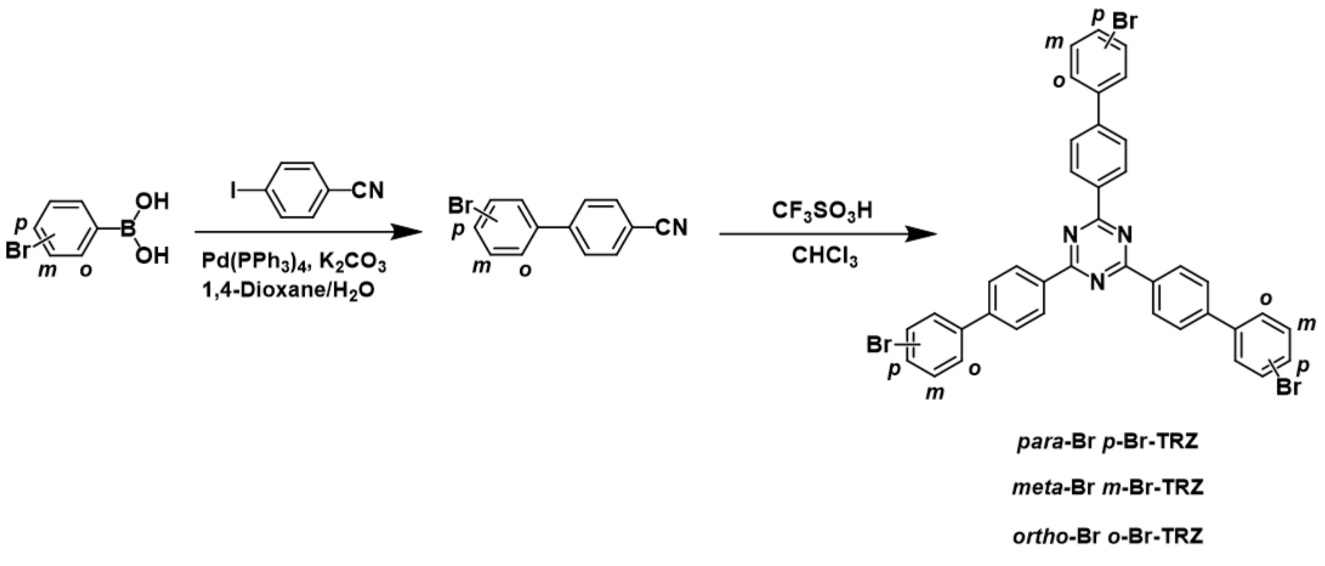

4'-bromo-(1,1'-biphenyl)-4-carbonitrile

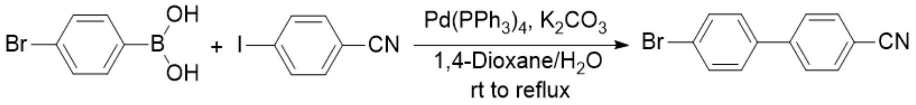

4-Bromophenylboronic acid (3.10 g, 14.9 mmol, purity 98%) was reacted with 4-iodobenzonitrile (4.07 g, 17.7 mmol, purity 98%) under Suzuki coupling reaction using $Pd(PPh_3)_4$ (261 mg, 0.23 mmol, purity 98%) and K_2CO_3 (purity 98%) aqueous solution (4.00 g, 29.7 mmol, 10 mL) as catalysts in 50 mL 1,4-dioxane (reflux under nitrogen). After cooling, water was added and the reaction mixture was extrated with CH_2Cl_2. The combined organic layer was washed with H_2O and brine, dried over anhydrous Na_2SO_4 (purity 99%), and evaporated under reduced pressure. The product was purified by column chromatography using petroleum ether/ethyl acetate (10:1 v/v) on silica gel to obtain a white solid as 4'-bromo-(1,1'-biphenyl)-4-carbonitrile (2.8 g, 70.3%). ^{1}H NMR (400 MHz, $CDCl_3$, δ/ppm): 7.73 (d, J = 8.0 Hz, 2H), 7.66–7.61 (m, 4H), 7.46 (d, J = 8.0 Hz, 2H). ^{13}C NMR (100 MHz, $CDCl_3$, δ/ppm) 144.4, 138.1, 132.7, 132.3, 128.8, 127.5, 123.2, 118.8, 111.3. MS (ESI): $[M+H]^+$ m/z calculated for $C_{13}H_9BrN$ 257.9913, found 257.9913.

3'-bromo-(1,1'-biphenyl)-4-carbonitrile

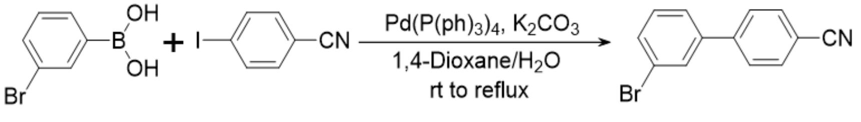

3'-bromo-(1,1'-biphenyl)-4-carbonitrile was synthesized according to 4'-bromo-(1,1'-biphenyl)-4-carbonitrile with 71.2% yield. ^{1}H NMR (600 MHz, $CDCl_3$, δ/ppm): 7.73–7.70 (m, 3H), 7.65–7.63 (m, 2H), 7.55–7.53 (m, 1H),

7.51–7.49 (m, 1H), 7.34 (t, J = 7.8 Hz, 1H); ^{13}C NMR (150 MHz, $CDCl_3$, δ/ppm) 144.1, 141.2, 132.7, 131.6, 130.7, 130.3, 127.8, 125.9, 123.3, 118.7, 111.6. MS (ESI): $[M+H]^+$ m/z calculated for $C_{13}H_9BrN$ 257.9913, found 257.9913.

2'-bromo-(1,1'-biphenyl)-4-carbonitrile

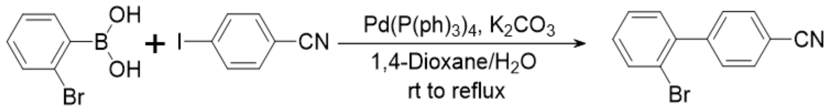

2'-bromo-(1,1'-biphenyl)-4-carbonitrile was synthesized according to 4'-bromo-(1,1'-biphenyl)-4-carbonitrile with 77.3% yield. ^{1}H NMR (400 MHz, DMSO, δ/ppm): 7.94 (d, J = 7.6 Hz, 2H), 7.78 (d, J = 8.0 Hz, 1H), 7.61 (d, J = 8.4 Hz, 2H), 7.50 (d, J = 7.2 Hz, 1H), 7.43–7.36 (m, 2H); ^{13}C NMR (150 MHz, $CDCl_3$, δ/ppm) 145.6, 140.7, 133.4, 131.9, 130.9, 130.3, 129.8, 127.7, 122.1, 118.8, 111.6. MS (ESI): $[M+H]^+$ m/z calculated for $C_{13}H_9BrN$ 257.9913, found 257.9913.

***p*-Br-TRZ**

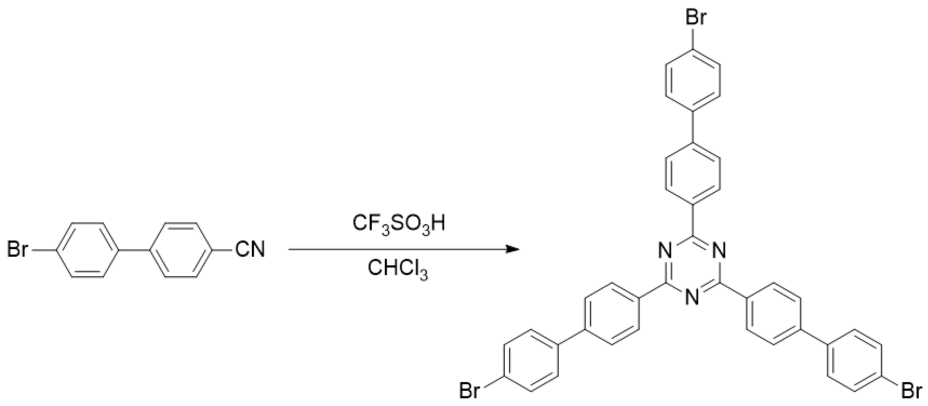

Trifluoromethanesulfonic acid (1.4 mL, 15.8 mmol, purity 99%) was dissolved in 5 mL of dry $CHCl_3$. The solution was stirred under ice bath, and then 4'-bromo-(1,1'-biphenyl)-4-carbonitrile (1.4 g, 7.5 mmol) in $CHCl_3$ (20 mL) was dropwise added. The mixture was kept at 0 °C for 1 h and then refluxed overnight. After cooling to room temperature, the mixture was quenched by aqua ammonia. The precipitate was filtered and washed by water, MeOH, CH_2Cl_2, respectively. The resultant white solid was collected as the target product 2,4,6-tris(4'-bromo-[1,1'-biphenyl]-4-yl)-1,3,5-triazine (*p*-Br-TRZ) (0.78 g, 56%). ^{1}H NMR (600 MHz, $CDCl_3$, δ/ppm) 8.81 (d, J = 10.2 Hz, 6H), 7.74 (d, J = 8.4 Hz, 6H), 7.62 (d, J = 8.4 Hz, 6H), 7.55 (d, J = 8.4 Hz, 6H); ^{13}C NMR (150 MHz, $CDCl_3$, δ/ppm) 171.3, 144.0, 139.3, 135.5, 132.1, 129.6, 128.8, 127.2, 122.4. MS (ESI): $[M+H]^+$ m/z calculated for $C_{39}H_{25}Br_3N_3$ 773.9578, found 773.9576. Elemental analysis (calcd., found for $C_{39}H_{24}Br_3N_3$): C (60.49, 60.26), N (5.43, 5.23), H (3.12, 3.16).

m-Br-TRZ

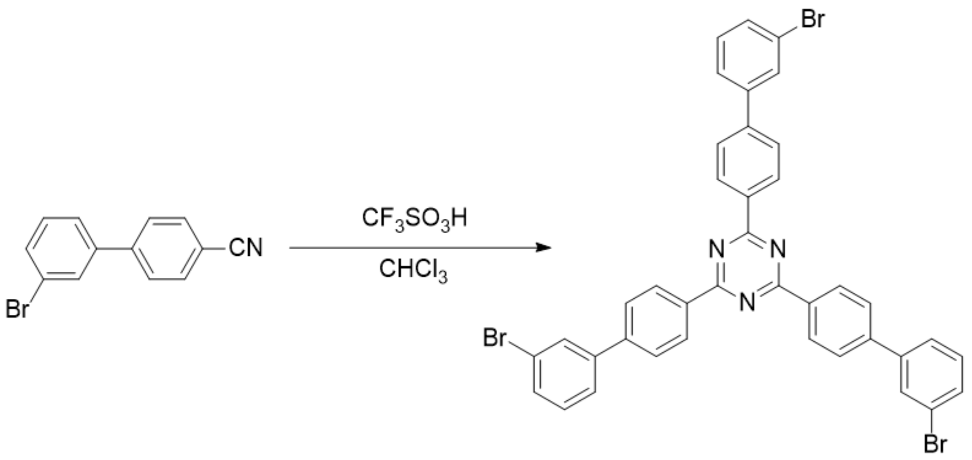

m-Br-TRZ was synthesized according to *p*-Br-TRZ with 78.6% yield. ^{1}H NMR (400 MHz, $CDCl_3$, δ/ppm): 8.89–8.84 (m, 6H), 7.88–7.82 (m, 6H), 7.79–7.77 (m, 3H), 7.64–7.52 (m, 6H), 7.39–7.34 (m, 3H); ^{13}C NMR (125 MHz, THF-d_8, δ/ppm) 171.3, 143.6, 142.5, 140.3, 135.7, 130.5, 130.0, 129.4, 127.1, 125.9, 122.8. MS (ESI): $[M+H]^+$ *m/z* calculated for $C_{39}H_{25}Br_3N_3$ 773.9578, found 773.9575. Elemental analysis (calcd., found for $C_{39}H_{24}Br_3N_3$): C (60.49, 60.41), N (5.43, 5.28), H (3.12, 3.16).

o-Br-TRZ

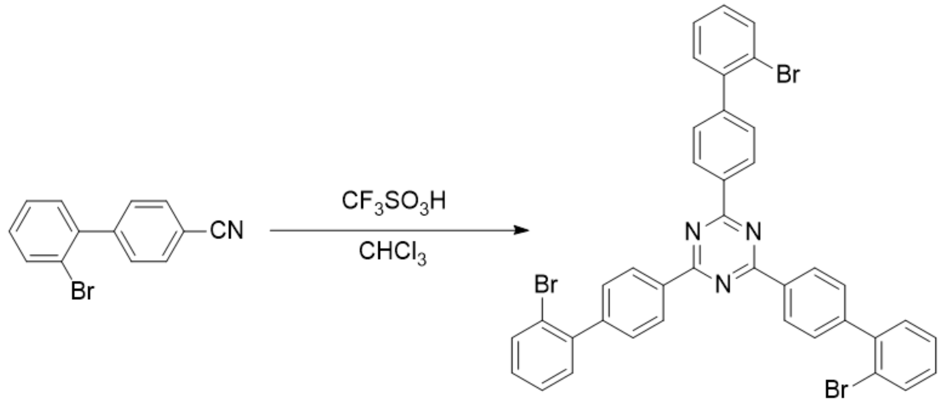

o-Br-TRZ was synthesized according to *p*-Br-TRZ with 61% yield. ^{1}H NMR (400 MHz, $CDCl_3$, δ/ppm): 8.73 (d, J = 8.4 Hz, 6H), 7.82 (d, J = 8.8 Hz, 6H), 7.76 (d, J = 8.0 Hz, 3H), 7.49–7.42 (m, 6H), 7.34–7.31 (m, 3H); ^{13}C NMR (100 MHz, $CDCl_3$, δ/ppm) 168.8, 150.0, 140.7, 133.5, 131.0, 130.9, 130.5, 130.1, 128.6, 127.8, 121.9. MS (ESI): $[M+H]^+$ *m/z* calculated for $C_{39}H_{25}Br_3N_3$ 773.9578, found 773.9576. Elemental analysis (calcd., found for $C_{39}H_{24}Br_3N_3$): C (60.49, 60.38), N (5.43, 5.43), H (3.12, 3.17).

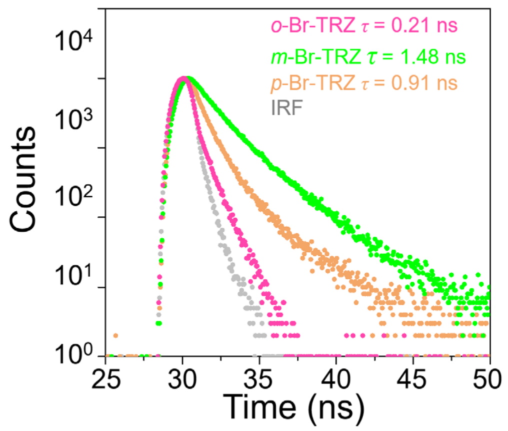

Supplementary Figure 1. Time resolved PL decay curves of *o*-Br-TRZ at 394 nm, *m*-Br-TRZ at 420 nm and *p*-Br-TRZ at 420 nm powders measured at room temperature. λ_{ex} = 320 nm.

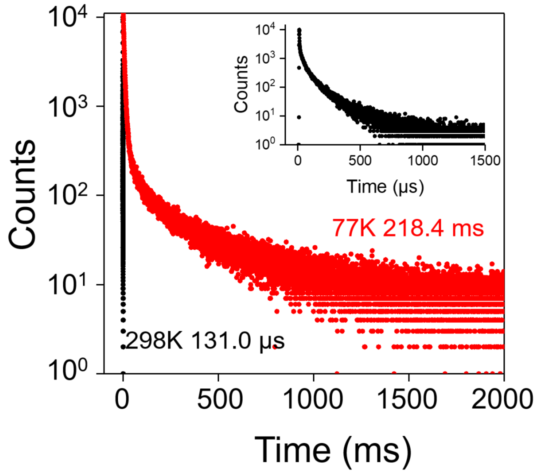

Supplementary Figure 2. Time resolved PL decay curves of *o*-Br-TRZ at 517 nm from 298 to 77 K. λ_{ex} = 350 nm.

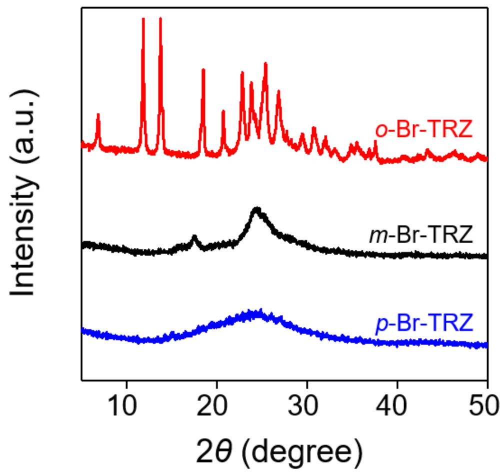

Supplementary Figure 3. X-ray diffraction of *o*-Br-TRZ, *m*-Br-TRZ, and *p*-Br-TRZ powders.

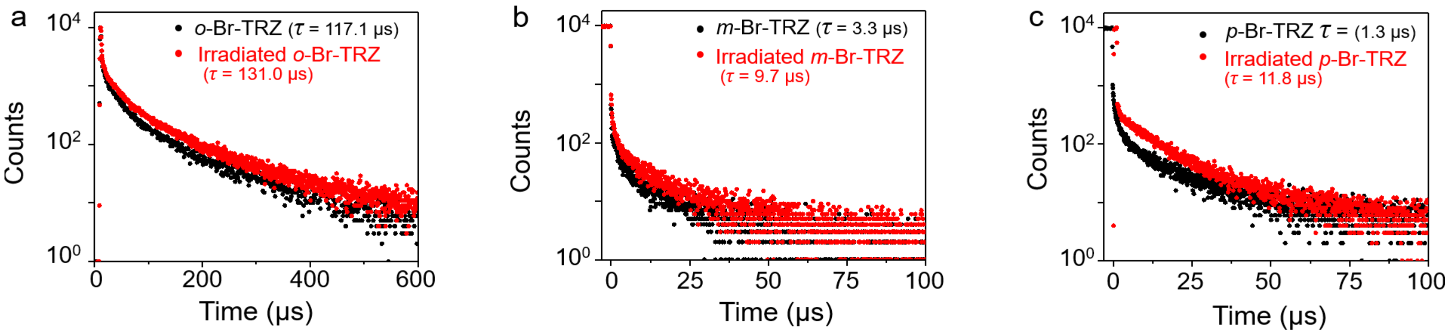

Supplementary Figure 4. Lifetime decay profiles of emission of (a) *o*-Br-TRZ at 527 nm, (b) *m*-Br-TRZ at 511 nm, and (c) *p*-Br-TRZ at 542 nm, before and after photoactivation under ambient conditions. λ_{ex} = 350 nm.

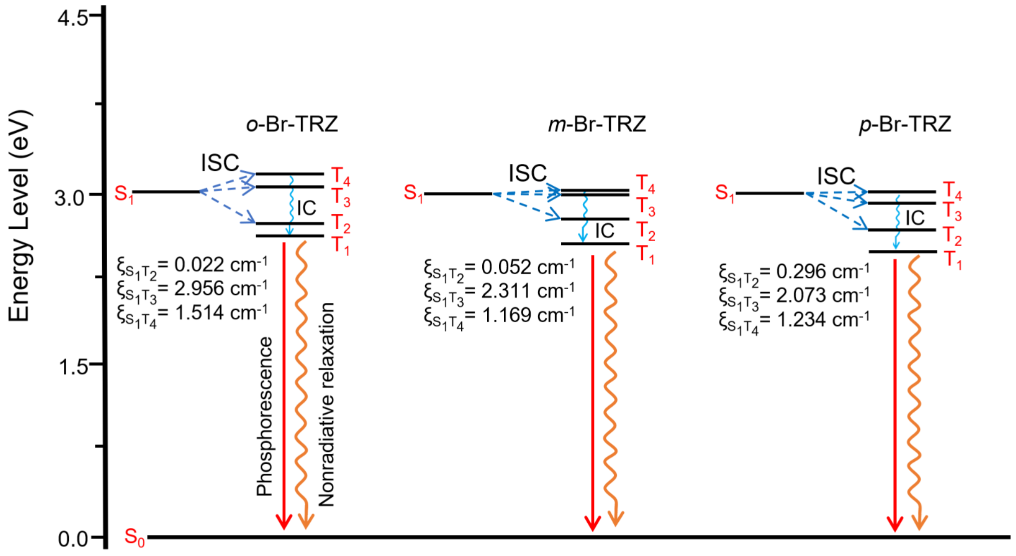

Supplementary Figure 5. Calculated energy diagram of S_1 and T_n, spin-orbit coupling for the involved S_1 and T_n states of *o*-Br-TRZ, *m*-Br-TRZ, and *p*-Br-TRZ.

Supplementary Table 1. The energy level and spin-orbit coupling of *o*-Br-TRZ, *m*-Br-TRZ, and *p*-Br-TRZ.

	S_1-T_n	ΔE_{ST} (eV)	ξ (cm^{-1})	ξ / exp (ΔE^2_{ST})
o-Br-TRZ	S_1-T_2	0.214	0.022	0.021
	S_1-T_3	0.067	2.956	2.944
	S_1-T_4	0.121	1.514	1.499
m-Br-TRZ	S_1-T_2	0.212	0.052	0.051
	S_1-T_3	0.064	2.311	2.302
	S_1-T_4	0.012	1.169	1.169
p-Br-TRZ	S_1-T_2	0.212	0.296	0.285
	S_1-T_3	0.103	2.073	2.052
	S_1-T_4	0.022	1.234	1.234

We performed theoretical calculations on these three compounds. According to the perturbation theory, the rate constant (k_{ISC}) of ISC is given by:

$k_{ISC} \propto \langle {}^1\Psi \, |\hat{H}_{SO}|\, {}^3\Psi \rangle / \exp(\Delta E^2_{ST})$ (1)

where $\langle {}^1\Psi \, |\hat{H}_{SO}|\, {}^3\Psi \rangle$ is the spin-orbit coupling (SOC, ξ) matix element, and ΔE_{ST} is the energy gap between the singlet and triplet states. This equation suggests that large SOC and small ΔE_{ST} can result in high k_{ISC}. Supplementary Fig. 18 shows that *o*-Br-TRZ, *m*-Br-TRZ and *p*-Br-TRZ all have three possible channels for ISC based on the same transition orbital compositions between T_n and S_1 within the ±0.3 eV energy level.[S1,S2] It is very coincident that all the $\xi_{S_1T_3}$ for *o*-Br-TRZ, *m*-Br-TRZ and *p*-Br-TRZ have the highest value with small ΔE_{ST}, becoming the most efficient ISC channel (Supplementary Fig. 18 and Table 1). The $\xi_{S_1T_3}/\exp(\Delta E^2_{S_1T_3})$ of *o*-Br-TRZ, *m*-Br-TRZ and *p*-Br-TRZ is 2.944, 2.302, and 2.052, respecitively, with small gaps. According to the equation 1 on k_{ISC}, the ISC rate constant is $k_{ISC(o\text{-Br-TRZ})} > k_{ISC(m\text{-Br-TRZ})} > k_{ISC(p\text{-Br-TRZ})}$ with small differences.

Theoretical and computational methods: All-electron density functional theory (DFT) calculations have been carried out by the latest version of ORCA quantum chemistry software[S3] (Version 5.0.1). For geometry optimization calculations of S_1 structure, wB97X-D3 functional and the def2-TZVP basis set[S4] were used, and the optimal S_1 geometry for each compound were determined. The DFT-D3 dispersion correction with BJ-damping[S5, S6] was applied to correct the weak interaction to improve the calculation accuracy. The excited states and Spin-Orbit Coupling (SOC) calculations were performed with PBE0 functional and the DKH-def2-TZVP basis set. SOC calculation was performed by spin-orbit mean-field (SOMF) method[S7]. To evaluate the torsion angle statistics between two benzene rings, long-range electrostatic interactions were accounted for using the Ewald method and the parameters for each like-site interaction were obtained using the COMPASS-II force field[S8].

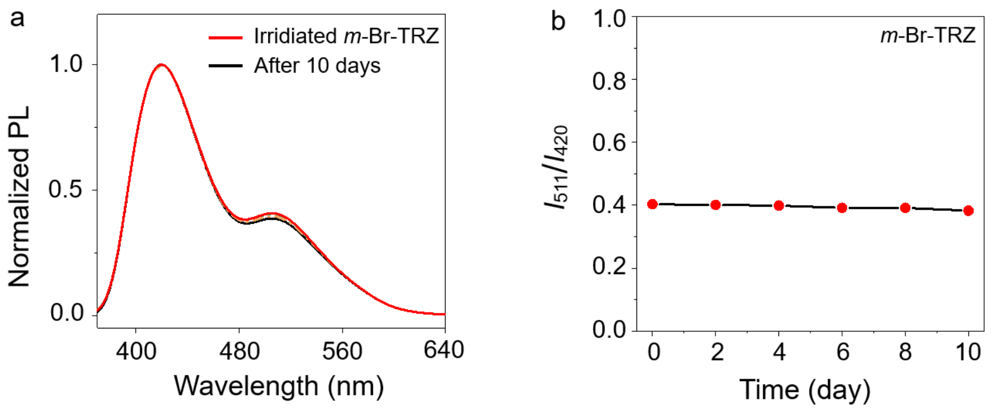

Supplementary Figure 6. (a) PL spectra of irradiation *m*-Br-TRZ before and after 10 days. (b) Luminescence intensity ratio (I_{511}/I_{420}), nearly no reversal change was observed after 10 days, indicating that irradiated *m*-Br-TRZ is stable.

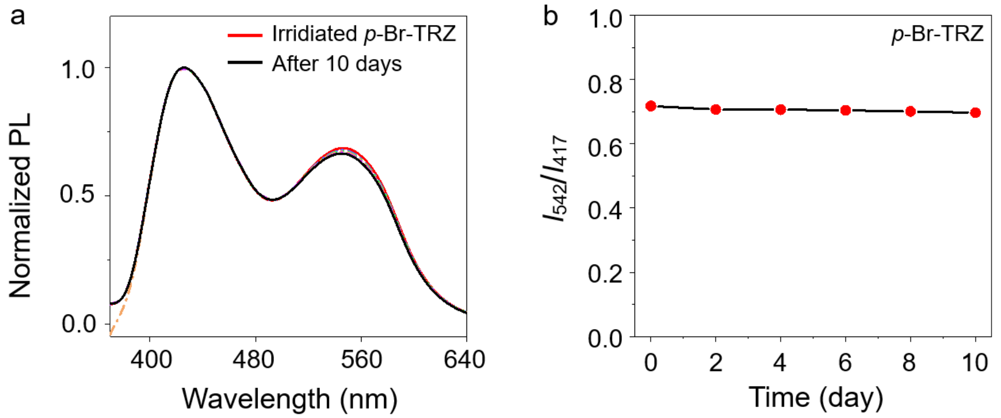

Supplementary Figure 7. (a) PL spectra of irradiation *p*-Br-TRZ before and after 10 days. (b) Luminescence intensity ratio (I_{542}/I_{417}), nearly no reversal change was observed after 10 days, indicating that irradiated *p*-Br-TRZ is stable.

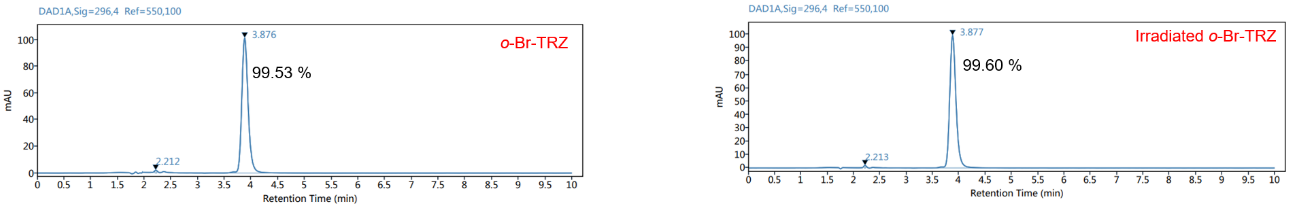

信号: DAD1A, Sig=296, 4 Ref=550, 100

保留时间 [min]	类型	峰宽 [min]	峰面积	高度	峰面积%	名称
2. 212	MM m	0. 03	4. 04	2. 12	0. 47	
3. 876	MB m	0. 13	847. 20	101. 45	99. 53	
		总和	851. 23			

信号: DAD1A, Sig=296, 4 Ref=550, 100

保留时间 [min]	类型	峰宽 [min]	峰面积	高度	峰面积%	名称
2. 213	MM m	0. 03	3. 34	1. 91	0. 40	
3. 877	MM m	0. 13	825. 81	99. 12	99. 60	
		总和	829. 14			

Supplementary Figure 8. HPLC spectra of *o*-Br-TRZ before and after 365 nm UV light irradiation for 90 s, indicating no change upon UV irradiation

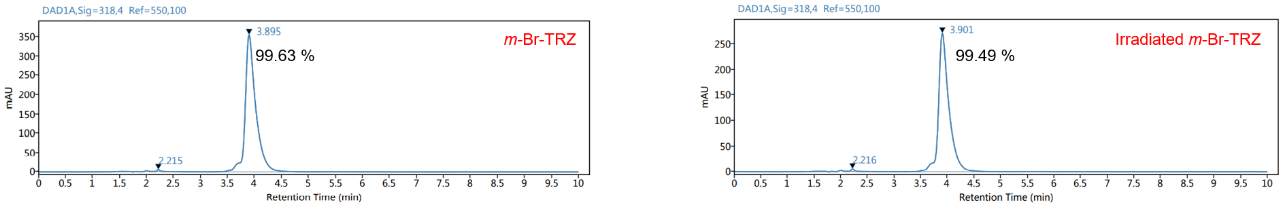

信号: DAD1A, Sig=318, 4 Ref=550, 100

保留时间 [min]	类型	峰宽 [min]	峰面积	高度	峰面积%	名称
2.215	MM m	0.06	18.02	4.73	0.37	
3.895	BB	2.04	4824.13	355.75	99.63	
		总和	4842.15			

信号: DAD1A, Sig-318, 4 Ref-550, 100

保留时间 [min]	类型	峰宽 [min]	峰面积	高度	峰面积%	名称
2.216	MM m	0.06	19.02	5.07	0.51	
3.901	BB	1.83	3687.20	271.76	99.49	
		总和	3706.22			

Supplementary Figure 9. HPLC spectra of *m*-Br-TRZ before and after 365 nm UV light irradiation for 90 s, indicating no change upon UV irradiation.

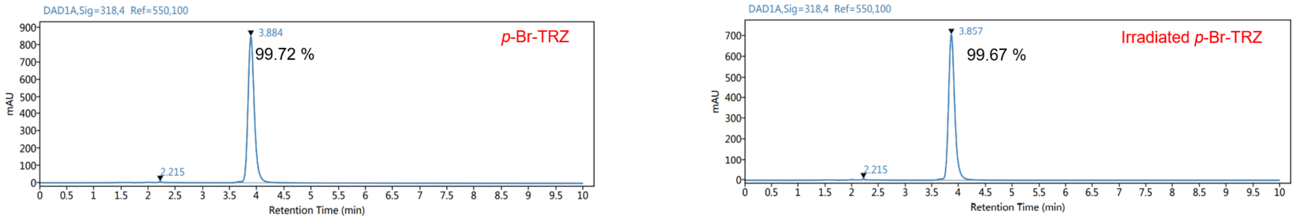

信号: DAD1A, Sig=318, 4 Ref=550, 100

保留时间 [min]	类型	峰宽 [min]	峰面积	高度	峰面积%	名称
2.215	MM m	0.06	19.31	5.15	0.28	
3.884	VM m	0.13	6973.12	850.64	99.72	
		总和	6992.43			

信号: DAD1A, Sig=318, 4 Ref=550, 100

保留时间 [min]	类型	峰宽 [min]	峰面积	高度	峰面积%	名称
2.215	MM m	0.06	19.21	5.17	0.33	
3.857	BM m	0.13	5779.40	707.17	99.67	
		总和	5798.60			

Supplementary Figure 10. HPLC spectra of *p*-Br-TRZ before and after 365 nm UV light irradiation for 90 s, indicating no change upon UV irradiation.

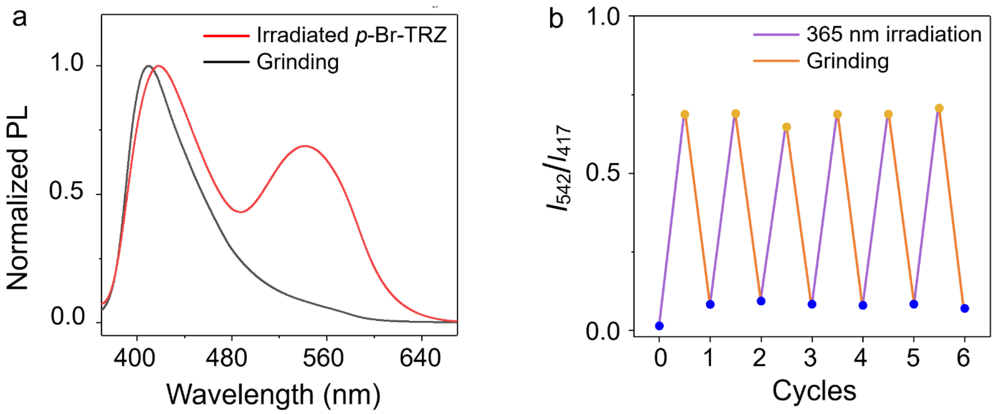

Supplementary Figure 11. (a) PL spectra of irradiated *p*-Br-TRZ before and after mechanical grinding. (b) Photo-induced RTP of *p*-Br-TRZ endows good fatigue resistance between photoirradiation and mechanical grinding.

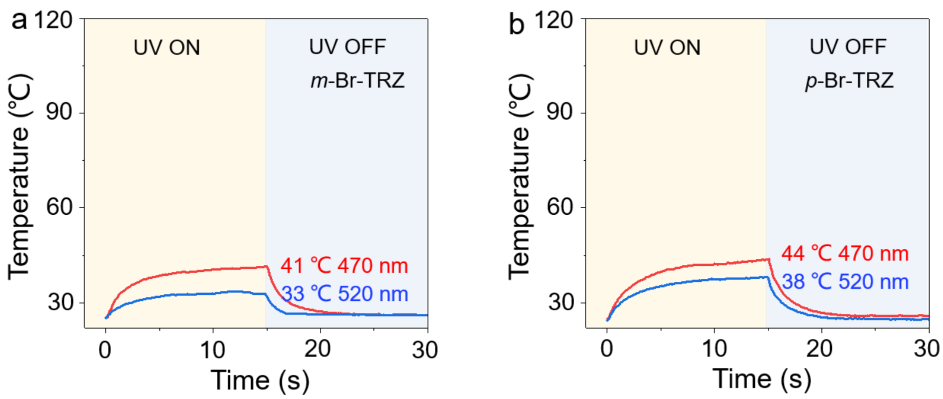

Supplementary Figure 12. Photothermal effects of (a) *m*-Br-TRZ and (b) *p*-Br-TRZ upon 470 nm (515 mW/cm^2) and 520 nm (512 mW/cm^2) irradiation.

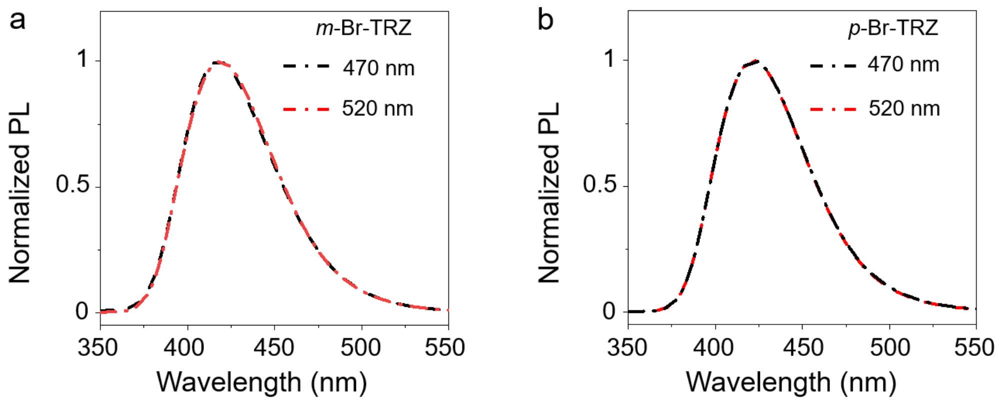

Supplementary Figure 13. PL spectra of (a) *m*-Br-TRZ and (b) *p*-Br-TRZ upon 470 (515 mW/cm^2) and 520 nm (512 mW/cm^2) irradiation for 90 s.

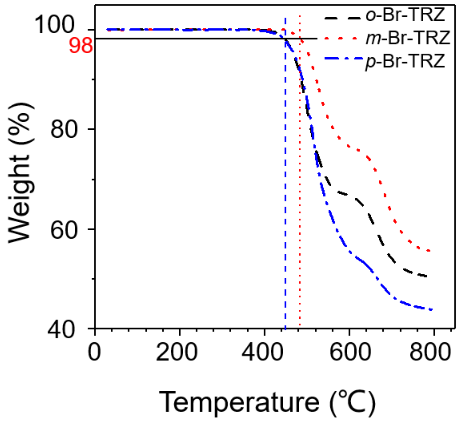

Supplementary Figure 14. TGA curves of *o*-Br-TRZ, *m*-Br-TRZ and *p*-Br-TRZ in N_2 at a heating rate of 20 °C/min. The 2% decomposition temperature of *o*-Br-TRZ and *p*-Br-TRZ is higher than 440 ^{o}C, while that of *m*-Br-TRZ is even higher than 480 ^{o}C, indicating all the three compounds have good thermal stability.

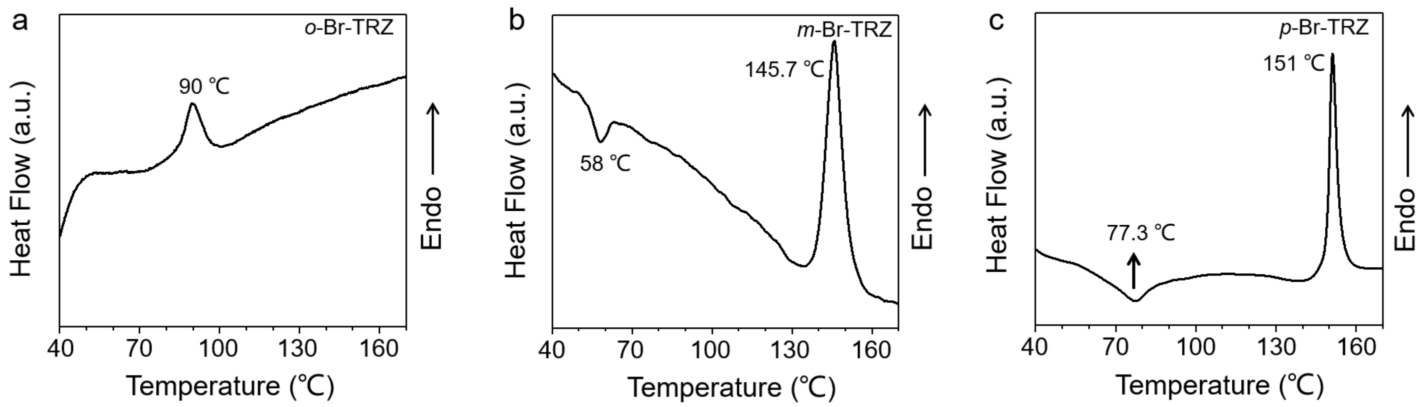

Supplementary Figure 15. DSC curves of (a) *o*-Br-TRZ, (b) *m*-Br-TRZ, and (c) *p*-Br-TRZ during heating at a heating rate of 10 °C/min. During heating, the exothermic transition at 58 and 77 °C, and endothermic transition at 146 and 151 °C for *m*-Br-TRZ and *p*-Br-TRZ are observed, respectively, which mean that there are intermolecular rearrangement processes ranging from 58 to 146 °C (*m*-Br-TRZ), and from 77 to 151 °C (*p*-Br-TRZ). For *o*-Br-TRZ, we only observed an endothermic transition at about 90 °C during heating.

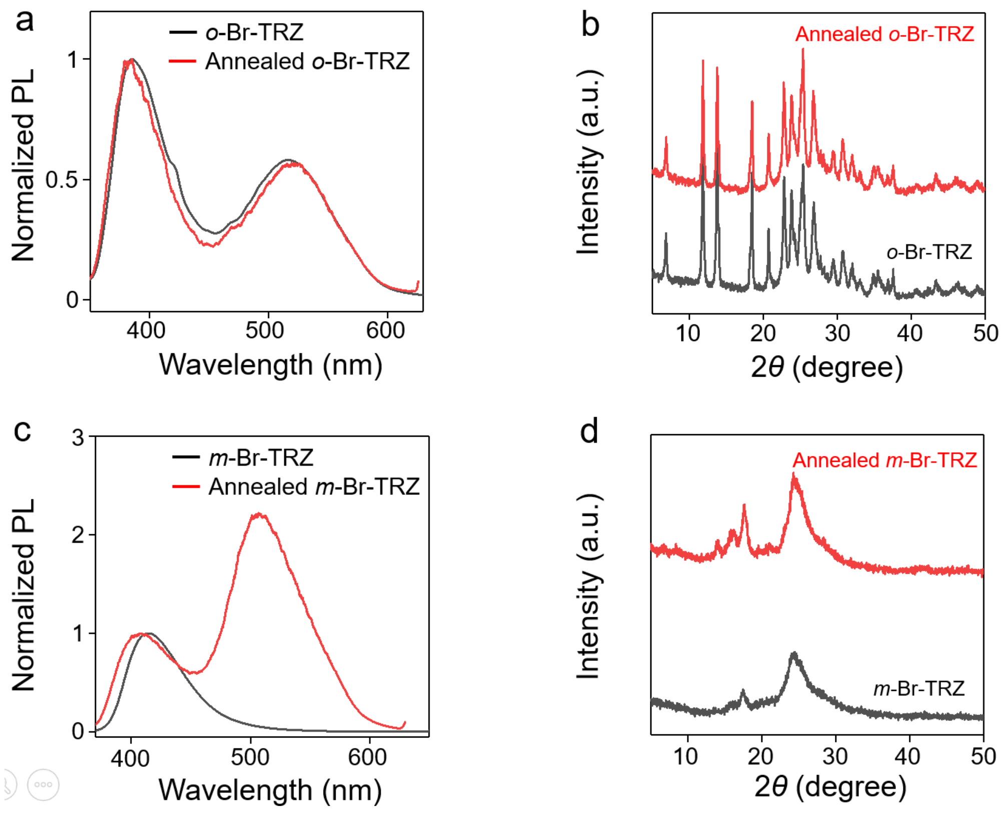

Supplementary Figure 16. (a) Normalized PL spectra of *o*-Br-TRZ after thermal annealing from 25 to 150 °C, and (b) its corresponding XRD. (c) Normalized PL spectra of *m*-Br-TRZ after thermal annealing from 25 to 150 °C, and (d) its corresponding XRD.

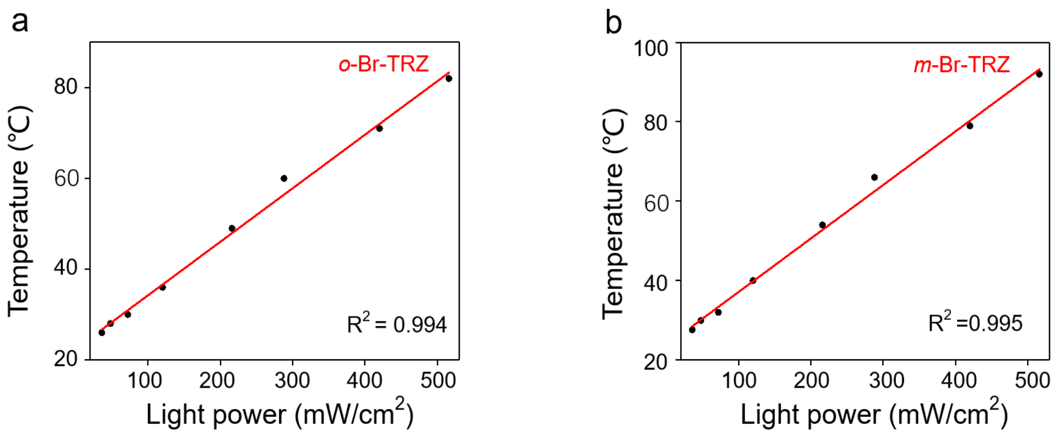

Supplementary Figure 17. Changes in the photothermal temperature of powdered *o*-Br-TRZ (a) and *m*-Br-TRZ (b) on increasing the power of 365 nm light from 0 to 516 mW/cm^2 demonstrate a linear relationship between photothermal temperature and irradiation powder.

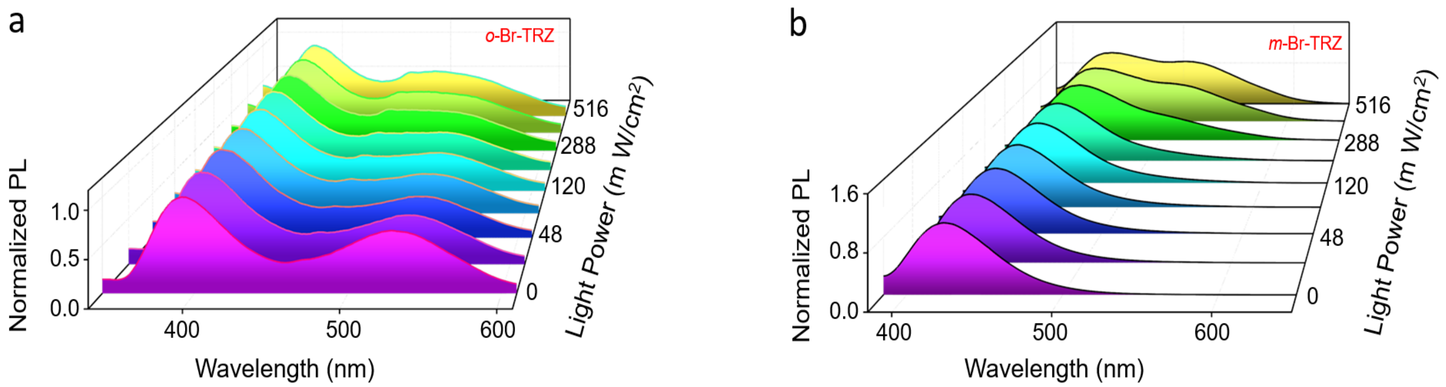

Supplementary Figure 18. Three-dimensional plot of luminescence of *o*-Br-TRZ (a) or *m*-Br-TRZ (b) vs power of 365 nm light vs wavelength showing dynamics of enhancement of phosphorescence and phosphorescence/fluorescence intensity ratio on increasing irradiation power.

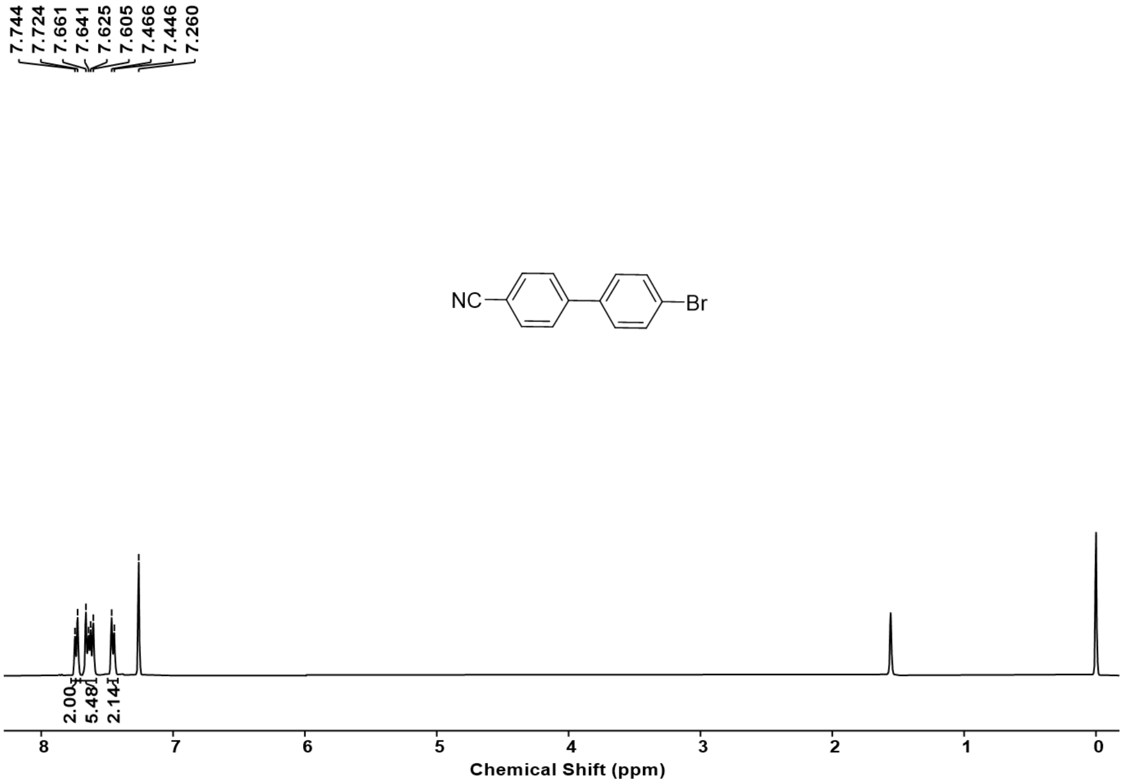

Supplementary Figure 19. ^{1}H NMR spectrum of 4'-bromo-(1,1'-biphenyl)-4-carbonitrile in $CDCl_3$.

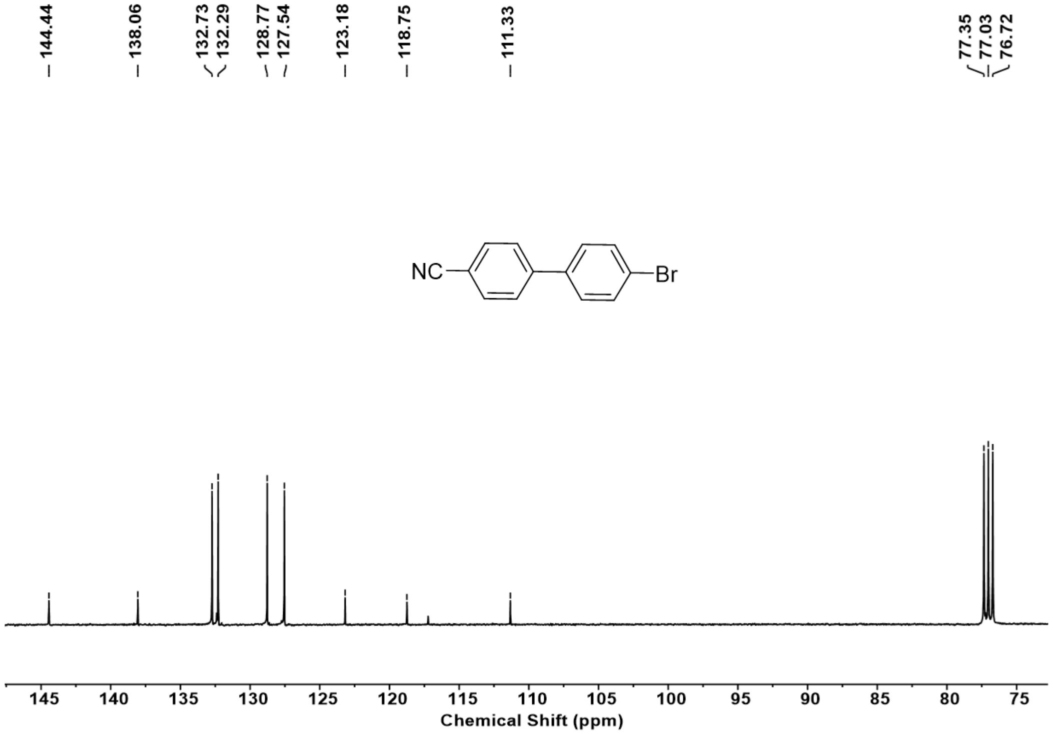

Supplementary Figure 20. ^{13}C NMR spectrum of 4'-bromo-(1,1'-biphenyl)-4-carbonitrile in $CDCl_3$.

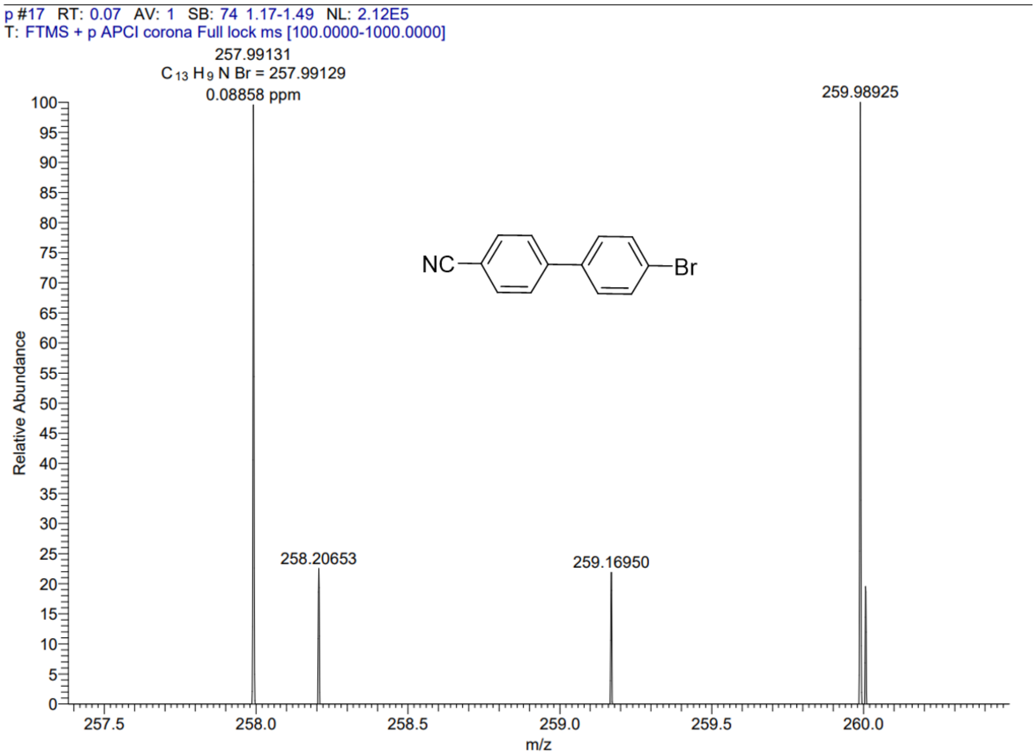

Supplementary Figure 21. ESI-MS spectrum of 4'-bromo-(1,1'-biphenyl)-4-carbonitrile.

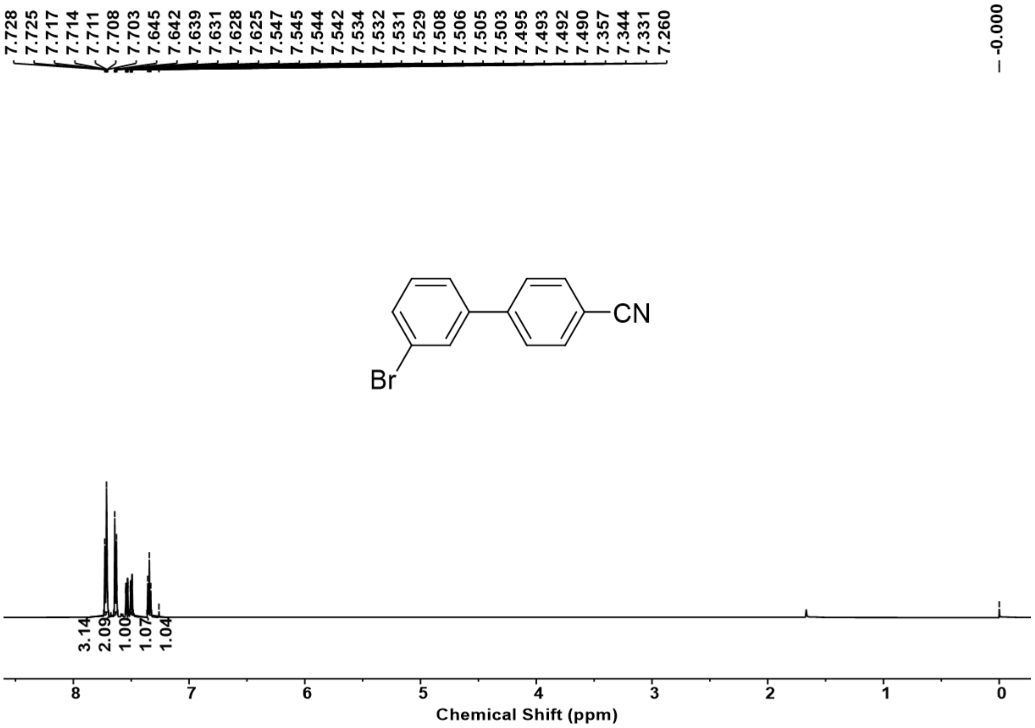

Supplementary Figure 22. ^{1}H NMR spectrum of 3'-bromo-(1,1'-biphenyl)-4-carbonitrile in $CDCl_3$.

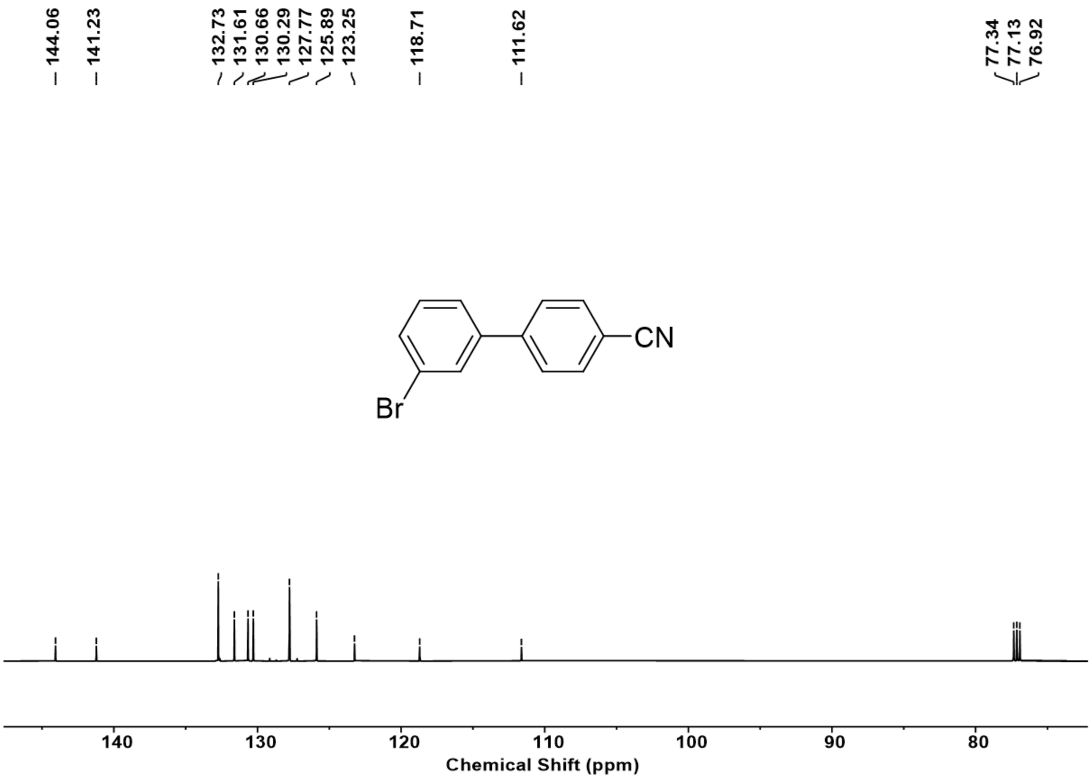

Supplementary Figure 23. ^{13}C NMR spectrum of 3'-bromo-(1,1'-biphenyl)-4-carbonitrile in $CDCl_3$.

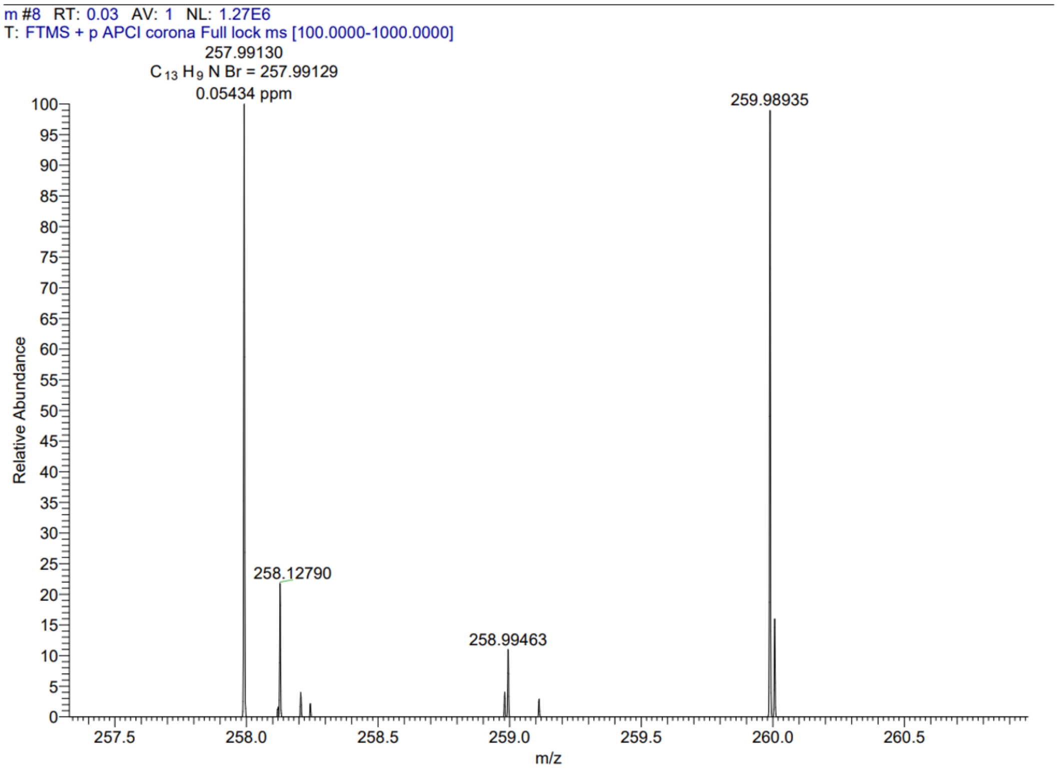

Supplementary Figure 24. ESI-MS spectrum of 3'-bromo-(1,1'-biphenyl)-4-carbonitrile.

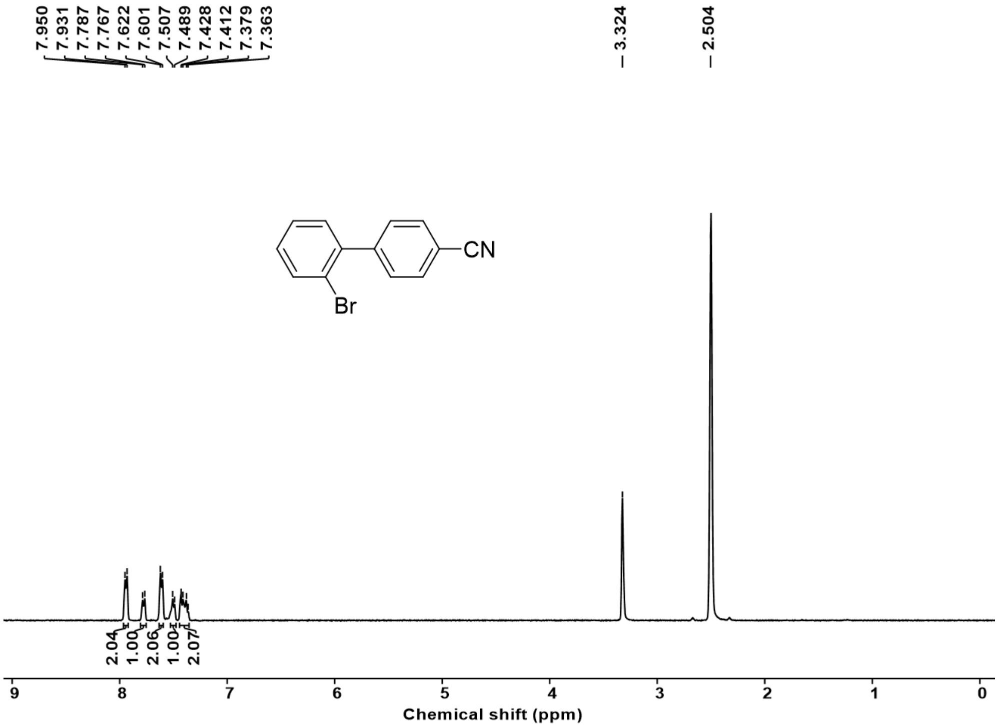

Supplementary Figure 25. ^{1}H NMR spectrum of 2'-bromo-(1,1'-biphenyl)-4-carbonitrile in DMSO.

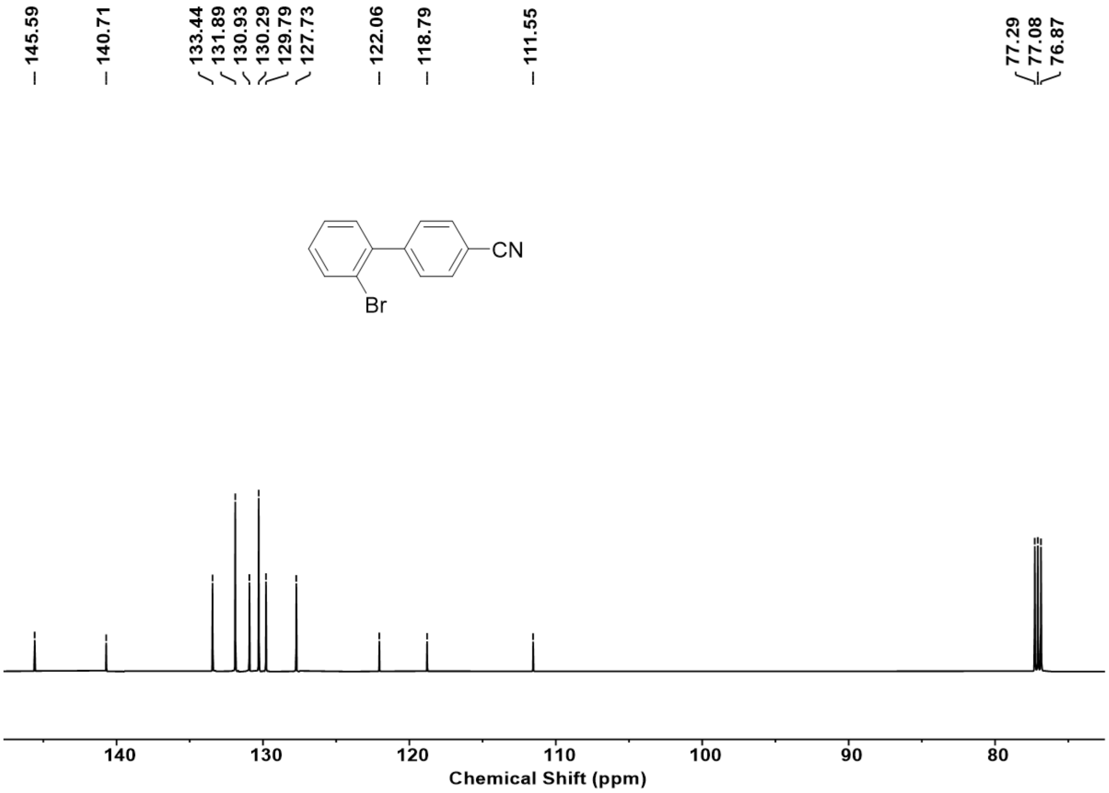

Supplementary Figure 26. ^{13}C NMR spectrum of 2'-bromo-(1,1'-biphenyl)-4-carbonitrile in $CDCl_3$.

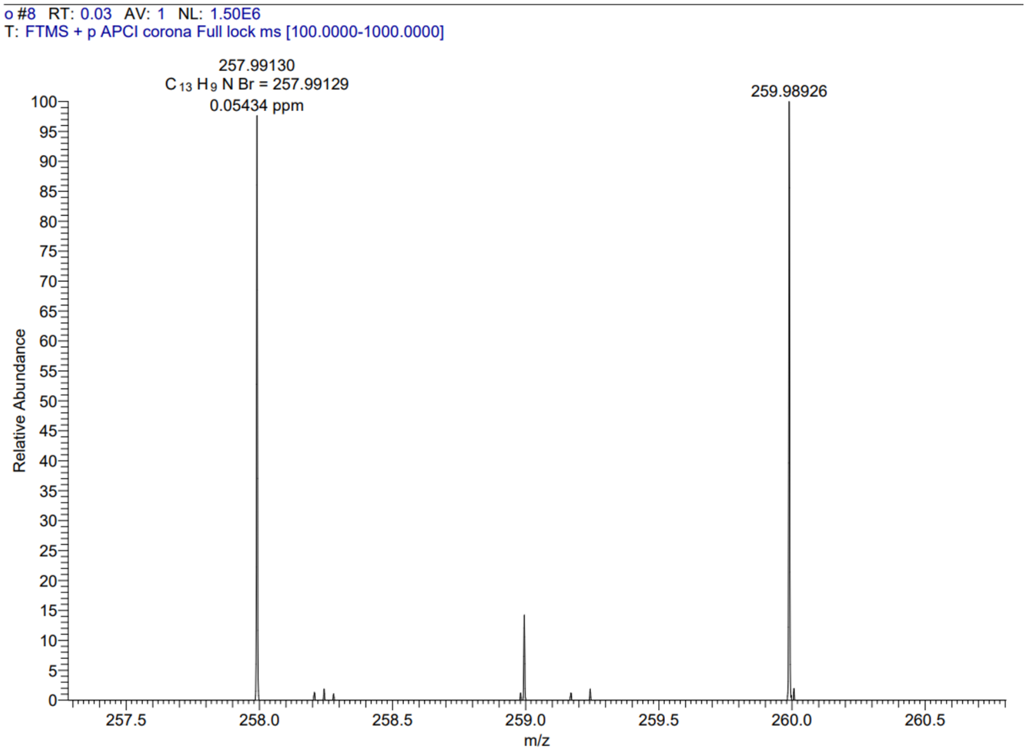

Supplementary Figure 27. ESI-MS spectrum of 2'-bromo-(1,1'-biphenyl)-4-carbonitrile.

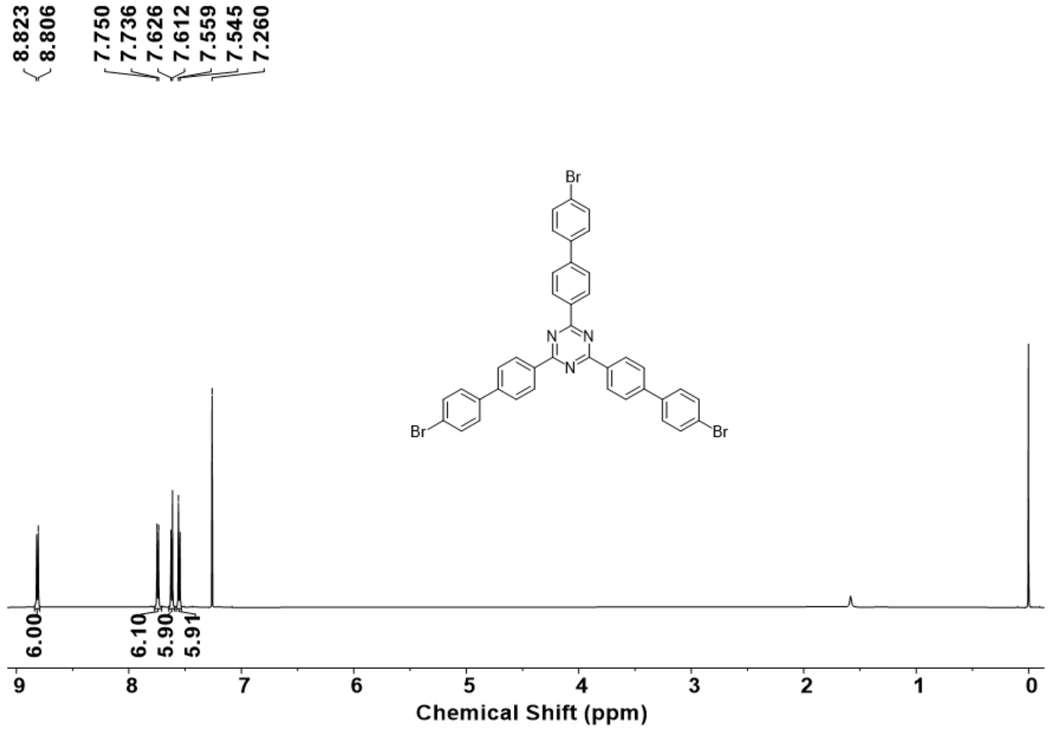

Supplementary Figure 28. ^{1}H NMR spectrum of *p*-Br-TRZ in $CDCl_3$.

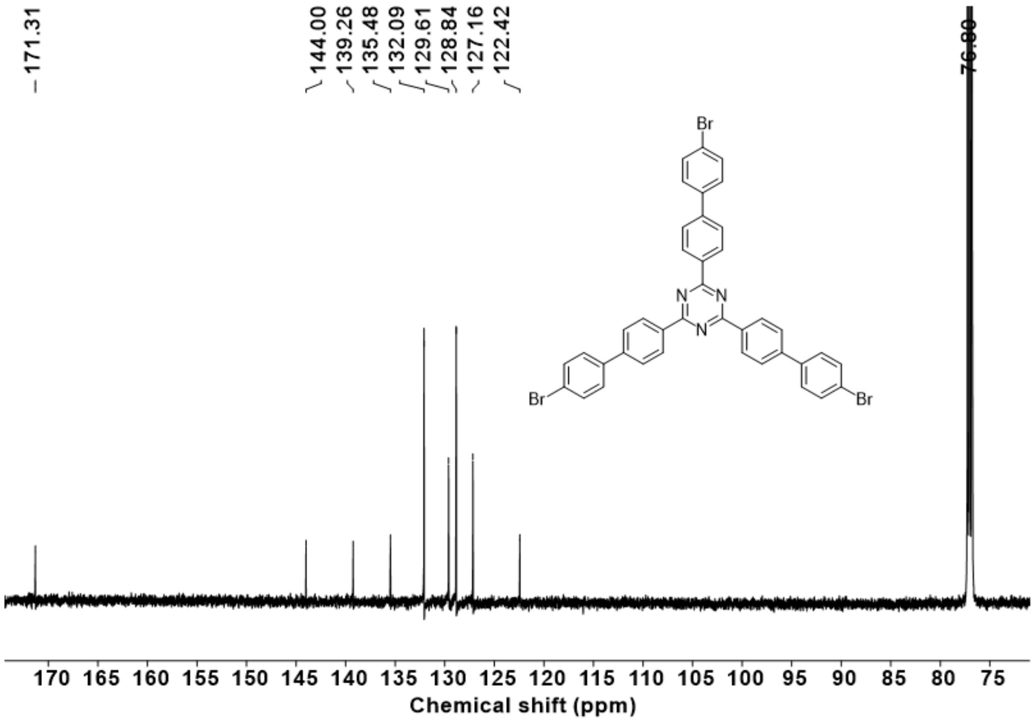

Supplementary Figure 29. ^{13}C NMR spectrum of *p*-Br-TRZ in $CDCl_3$.

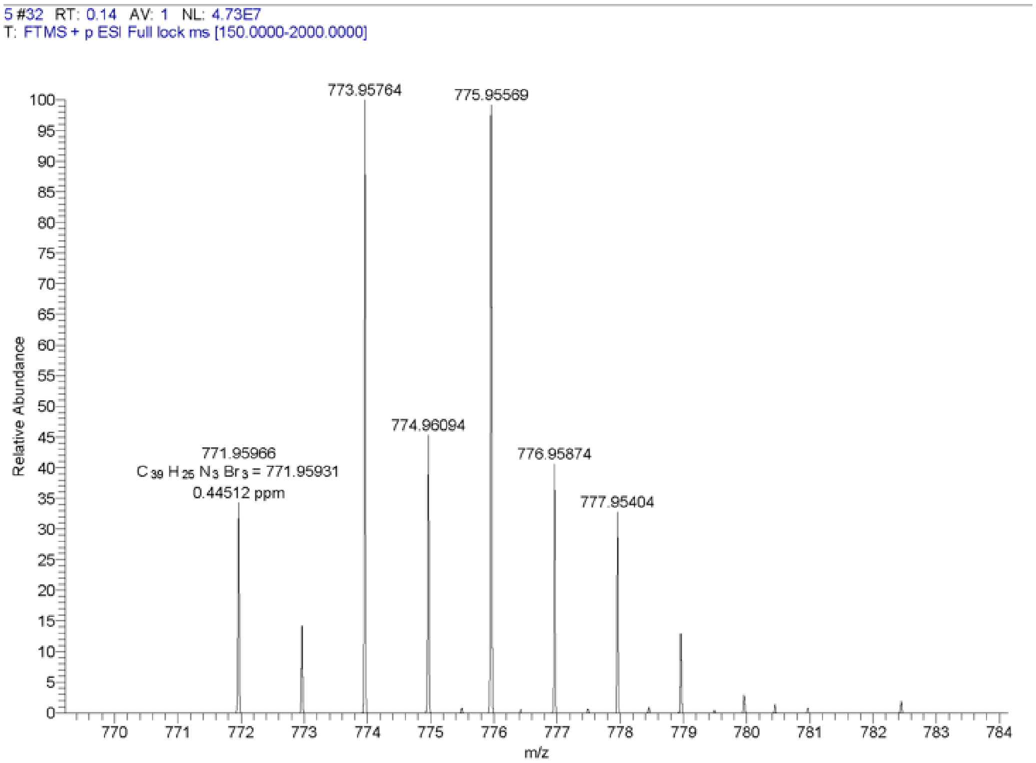

Supplementary Figure 30. ESI-MS spectrum of *p*-Br-TRZ.

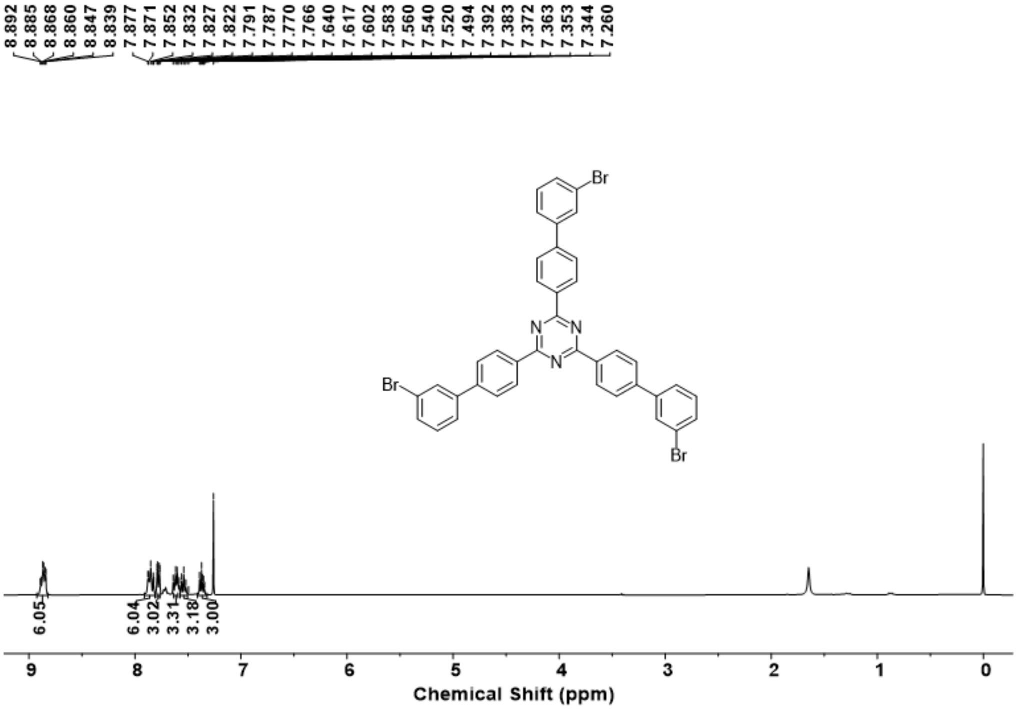

Supplementary Figure 31. ^{1}H NMR spectrum of *m*-Br-TRZ in $CDCl_3$.

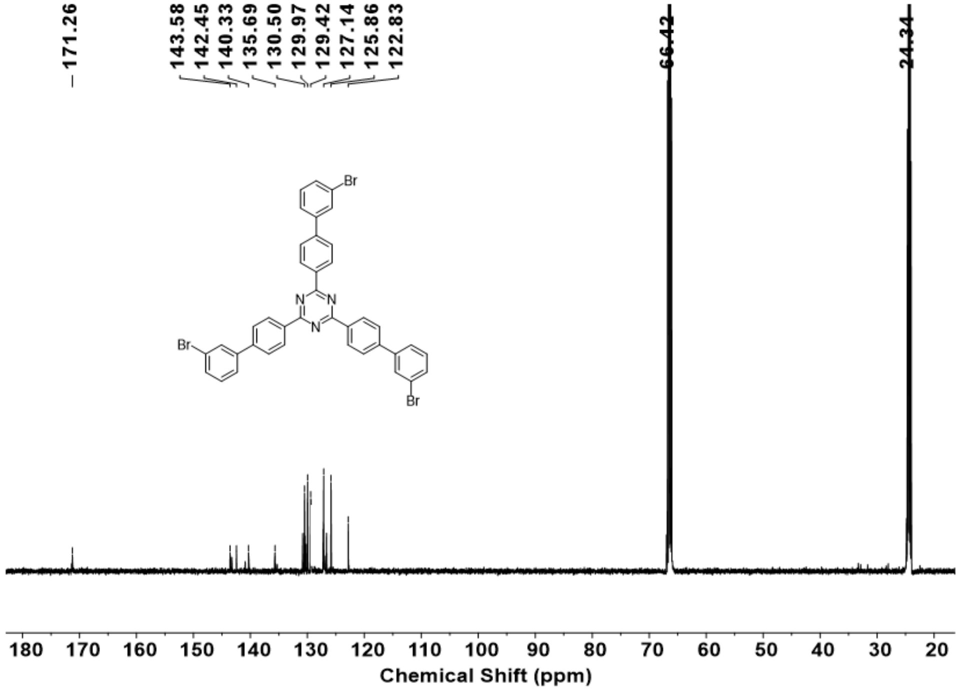

Supplementary Figure 32. ^{13}C NMR spectrum of *m*-Br-TRZ in THF-d_8.

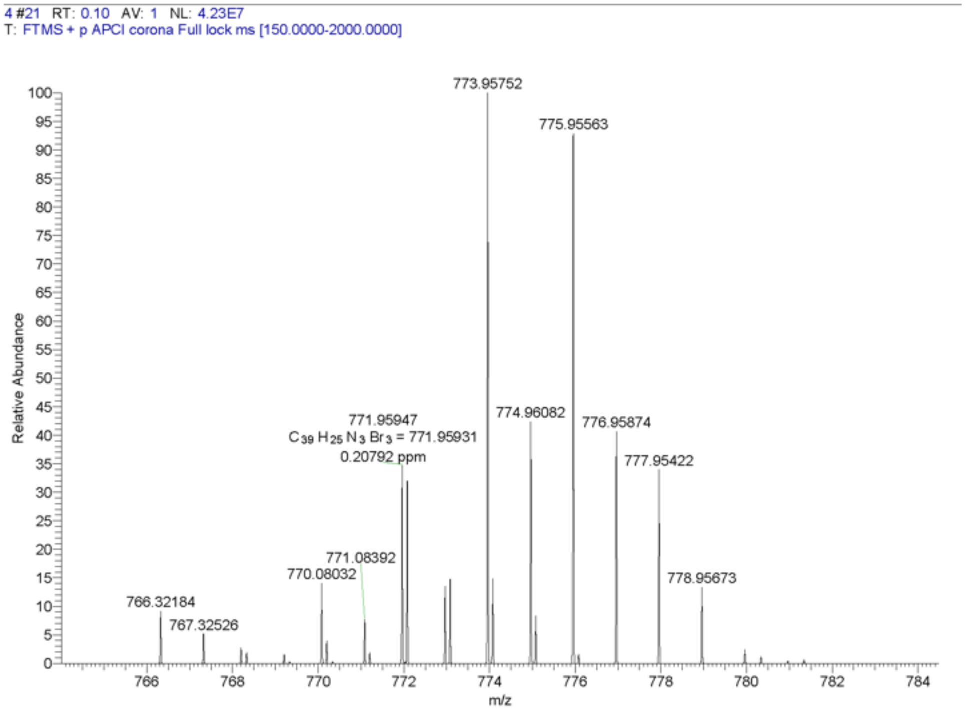

Supplementary Figure 33. ESI-MS spectrum of *m*-Br-TRZ.

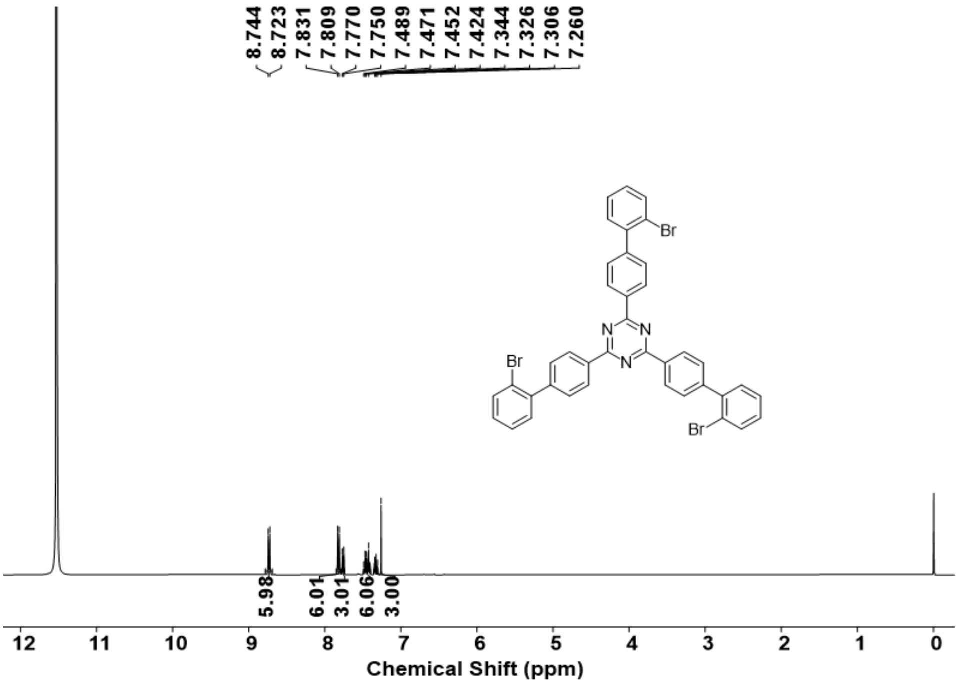

Supplementary Figure 34. ^{1}H NMR spectrum of *o*-Br-TRZ in $CDCl_3$. Note: we added trifluoroacetic acid for better solubility of *o*-Br-TRZ in $CDCl_3$.

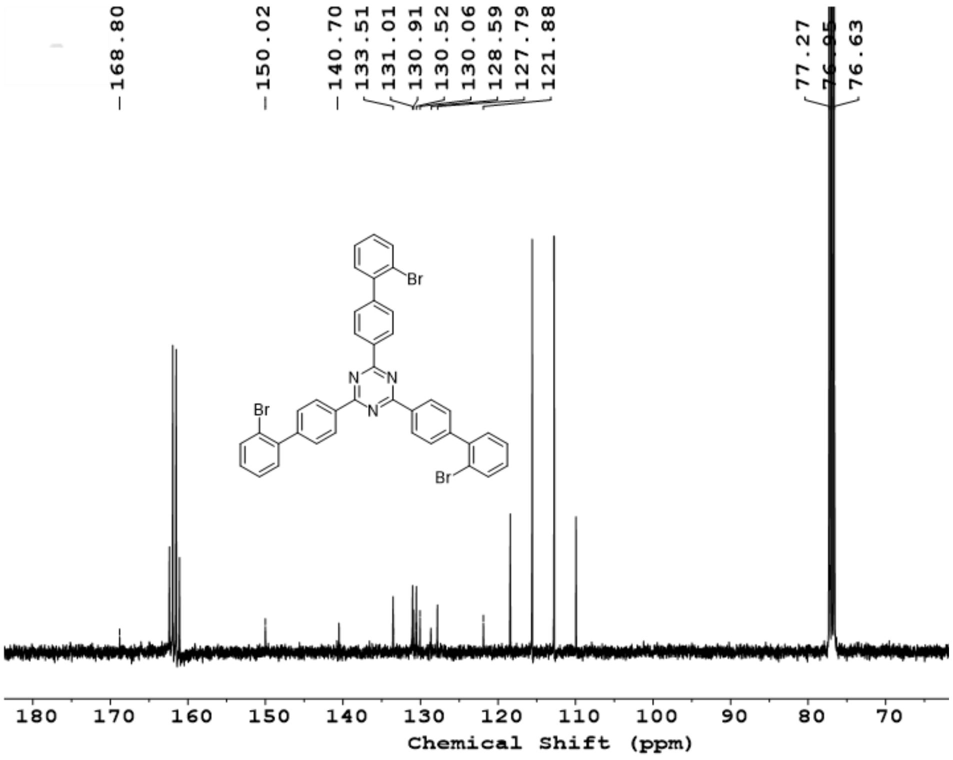

Supplementary Figure 35. ^{13}C NMR spectrum of *o*-Br-TRZ in $CDCl_3$. Note: we added trifluoroacetic acid for better solubility of *o*-Br-TRZ in $CDCl_3$.

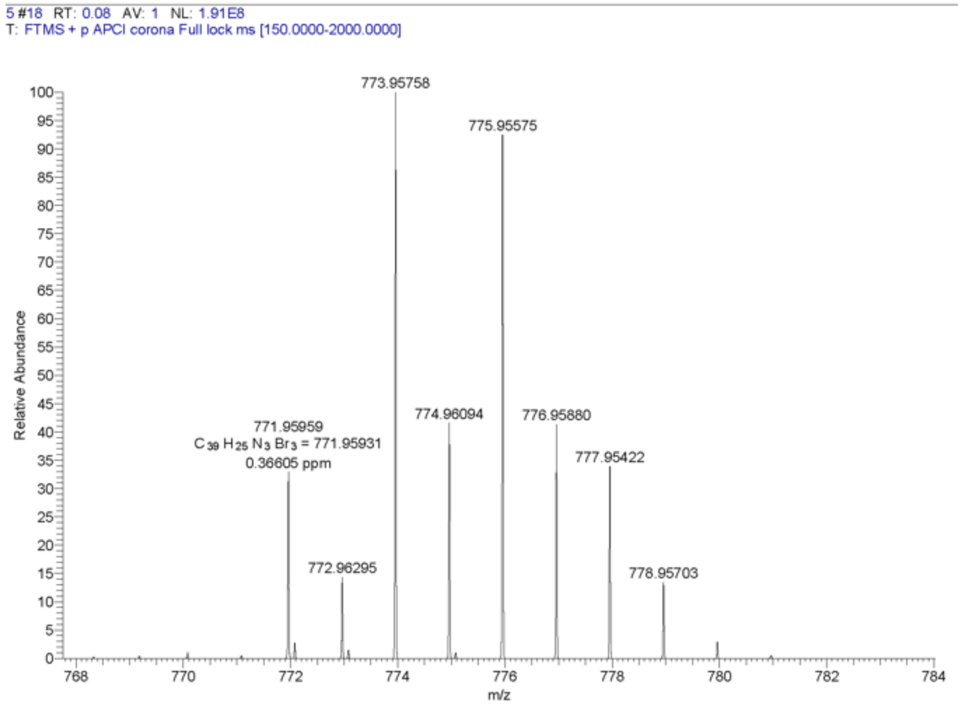

Supplementary Figure 36. ESI-MS spectrum of *o*-Br-TRZ.

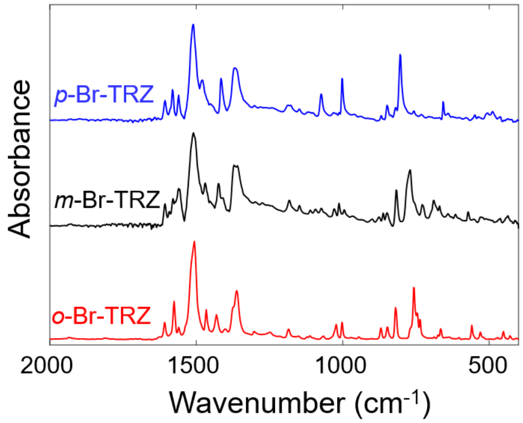

Supplementary Figure 37. Fourier transform infrared (FTIR) spectra of *o*-Br-TRZ, *m*-Br-TRZ and *p*-Br-TRZ.

The FTIR spectra of the three isomers are shown in Supplementary Figure 37. The in-plane stretching vibrations of aryl sym-triazines occur at 1580-1520 cm^{-1} and 1450-1350cm^{-1}. In the FTIR spectra of the compounds, two peaks can be found near 1560 and 1425 cm^{-1}. We deduced the bands belong to triazine. in Supplementary Figure 37, the three peaks around 1607, 1580, and 1507 cm^{-1} belong to the ring carbon-carbon stretching vibrations. The absorption band near 1507 cm^{-1} is the characteristic peak of the aromatic skeletal vibration and is the strongest band. Due to the conjugated effect, a doublet of ring carbon-carbon stretching vibrations can be observed at 1616-1570 cm^{-1} and the band near 1580 cm^{-1} is stronger than the peak around 1607 cm^{-1}. The above information confirms the existence of substituted benzenes. Moreover, the peak around 1072 cm^{-1} is the C-Br stretching vibration in the meta- or para-position of substituted benzenes. The peak around 1022 cm^{-1} is the C-Br stretching vibration in the ortho-position of substituted benzenes.

References

S1. Fraser, C. *et, al*. Multi-Emissive Difluoroboron Dibenzoylmethane Polylactide Exhibiting Intense Fluorescence and Oxygen-Sensitive Room-Temperature Phosphorescence. *J. Am. Chem. Soc.* **129**, 8942-8943 (2007).

S2. Bunz, U. *et, al*. Twisted Tethered Tolanes: Unanticipated Long-Lived Phosphorescence at 77 K. *J. Am. Chem. Soc.* **135**, 2160–2163 (2013).

S3. Neese, F. Software update: the ORCA program system, version 4.0. *Computational Molecular Science*. **8**: e1327 (2018).

S4. Weigend, F. & Ahlrichs, R. Balanced basis sets of split valence, triple zeta valence and quadruple zeta valence quality for H to Rn: Design and assessment of accuracy. *Phys. Chem. Chem. Phys*. **7**, 3297-3305 (2005).

S5. Goerigk, L. *et al*. Effect of the Damping Function in Dispersion Corrected Density Functional Theory. *J Comput Chem*, **32**, 1456–1465 (2011).

S6. Krieg, H. *et al.* A consistent and accurate ab initio parametrization of density functional dispersion correction (DFT-D) for the 94 elements H-Pu. *J.Chem.Phys*. **132**, 154104 (2010).

S7. Gropen, O. *et al.* A mean-field spin-orbit method applicable to correlated wavefunctions. *Chem. Phys. Lett*. **251**, 365-371 (1996).

S8. Todd, S. *et al.* COMPASS II: extended coverage for polymer and drug-like molecule databases. *J Mol Model.* **22**: 47 (2016).
